# Impact of the mouse model and molar amount of injected ligand on the tissue distribution profile of PSMA radioligands

**DOI:** 10.1007/s00259-021-05446-5

**Published:** 2021-08-17

**Authors:** Viviane J. Tschan, Francesca Borgna, Roger Schibli, Cristina Müller

**Affiliations:** 1 Center for Radiopharmaceutical Sciences, ETH-PSI-USZ, Paul Scherrer Institute, 5232 Villigen-PSI, Switzerland; 2 Department of Chemistry and Applied Biosciences, ETH Zurich, 8093 Zurich, Switzerland

**Keywords:** PSMA ligands, Albumin binder, PC-3 PIP, LNCaP, Molar amount

## Abstract

**Purpose:**

Various preclinical study designs are described in the literature for the evaluation of PSMA radioligands. In this study, [^177^Lu]Lu-Ibu-DAB-PSMA, an albumin-binding radioligand, and [^177^Lu]Lu-PSMA-617 were investigated and compared under variable experimental conditions.

**Methods:**

In vitro cell uptake studies were performed with PC-3 PIP and LNCaP tumor cells using a range of molar concentrations (0.75–500 nM) of both radioligands. Biodistribution and SPECT/CT imaging studies were carried out with the respective tumor mouse models using 0.05 nmol and 1.0 nmol injected ligand per mouse.

**Results:**

In both tumor cell lines, the uptake of the radioligands was increased when using low molar concentrations of the respective ligand. The observed saturation effect at high ligand concentrations was more pronounced for LNCaP cells that express PSMA at lower levels than for PC-3 PIP cells. At all investigated timepoints, the in vivo uptake of both radioligands was higher in PC-3 PIP tumors than in LNCaP tumors. A low molar amount of injected ligand increased the PC-3 PIP tumor uptake mainly for [^177^Lu]Lu-Ibu-DAB-PSMA; however, the molar amount of ligand was relevant for both radioligands when using LNCaP tumors. Renal retention of both radioligands was, however, up to fourfold higher during the first hours after application of a low ligand amount compared to the high ligand amount.

**Conclusion:**

The results of this preclinical study underline the relevance of the tumor model and applied ligand amount for the characterization of PSMA radioligands. The application of equal preclinical study designs is crucial to allow the comparison of novel radioligands with existing ones and, thus, predict potential advantages of new radioligands in view of a clinical application.

**Supplementary Information:**

The online version contains supplementary material available at 10.1007/s00259-021-05446-5.

## Introduction

The evaluation of novel radiopharmaceuticals relies on a variety of in vitro and in vivo experiments, which enable their detailed characterization. The applied protocols for the preclinical studies vary, however, considerably between different laboratories. This situation makes the comparability between new and existing radiopharmaceuticals often challenging or even impossible. While the tumor mouse model is an important determinant for the resultant data, it is in particular the molar amount of injected ligand that was previously shown to have an impact on the biodistribution of different peptide-based radiopharmaceuticals [1–4]. Indeed, depending on the choice of these parameters, the overall picture of the radioligand’s properties may vary and lead to controversial conclusions.

Prostate-specific membrane antigen (PSMA)-targeting radioligands have shown promising results for imaging and therapy of metastatic castration-resistant prostate cancer (mCRPC) [5–8]. Several novel PSMA radioligands are currently under preclinical development [9]. Comparison of the data from different research groups appears challenging, in particular because of different cell lines and xenograft models that are used for the evaluation of the radioligands. The two predominantly used prostate cancer cell lines are PC-3 PIP tumor cells—commonly combined with PC-3 flu tumor cells as a PSMA-negative control—and LNCaP tumor cells [10–13]. The PC-3 PIP cell line is transduced with PSMA [14, 15] and characterized with a high expression level (~ 4.9 × 10^6^ receptors/cell), whereas the LNCaP cell line expresses PSMA naturally, but at much lower levels (~ 5.9 × 10^5^ receptors/cell) [16, 17]. Due to these differences, the comparison of new PSMA radioligands evaluated in either PC-3 PIP or LNCaP tumor-bearing mice [17–19] is not possible, as the tumor accumulation depends on the xenograft type. The second critical aspect refers to the molar amount of injected ligand per mouse, which varies among the diverse studies from picomolar up to nanomolar quantities [17–19]. This can lead to variable degrees of receptor saturation in PSMA-expressing tissue and, possibly also affect the distribution in PSMA-negative organs. As an example, it is referred to the evaluation of novel albumin-binding radioligands by Kelly et al. [20] and Deberle et al. [21] who tested [^177^Lu]Lu-RPS-072, injected at 13–23 pmol in male LNCaP tumor-bearing mice and [^177^Lu]Lu-Ibu-DAB-PSMA, applied at 1.0 nmol in female PC-3 PIP tumor-bearing mice, respectively.

**Fig. 1:**
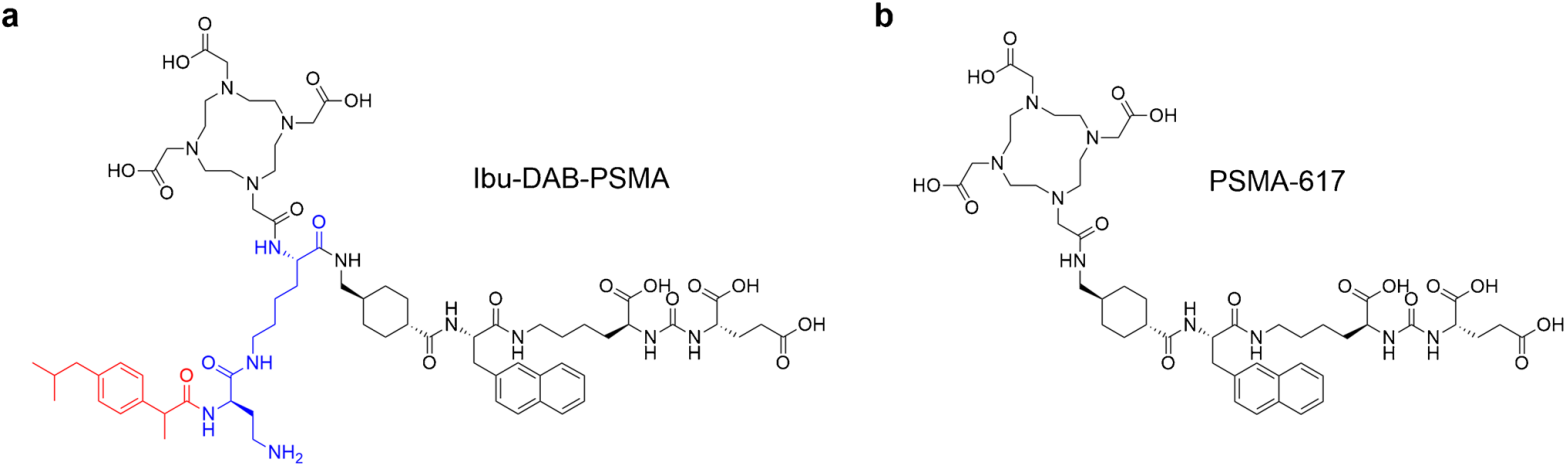
**a** Chemical structure of Ibu-DAB-PSMA, modified with ibuprofen (red) as an albumin-binding entity conjugated via a lysine-d-diaminobutyric acid (DAB) linker entity (blue) [21]; **b** Chemical structure of PSMA-617 [22]

In this study, we set out to investigate the impact of the xenograft model (PC-3 PIP versus LNCaP) and the molar amount of applied ligand on the in vitro and in vivo characterization of PSMA radioligands. For this purpose, we used [^177^Lu]Lu-Ibu-DAB-PSMA, an albumin-binding PSMA radioligand developed in our own group [21], and [^177^Lu]Lu-PSMA-617 [22], the current “gold-standard” that is being tested in a Phase III clinical study (VISION; NCT03511664) (Fig. 1) [23]. The selection of these two radioligands allowed exploring whether longer circulating PSMA radioligands would show a different response to changes in the study design than conventional, fast-cleared radioligands.

## Materials and methods

### Radiolabeling

The radiolabeling of Ibu-DAB-PSMA and PSMA-617 was performed under standard labeling conditions at pH 4.5 using lutetium-177 (no-carrier-added [^177^Lu]LuCl_3_ in 0.04 M HCl; ITM Medical Isotopes GmbH, Germany) as previously reported [17, 21]. Quality control using HPLC revealed ≥ 95% radiochemical purity of the radioligands prepared at molar activities between 5 and 150 MBq/nmol. The radioligands were used for in vitro and in vivo experiments without further purification.

### Cell culture

Sublines of the androgen-independent PC-3 human prostate cancer cell line, PSMA-positive PC-3 PIP and PSMA-negative PC-3 flu cells, were kindly provided by Prof. Dr. Martin Pomper (Johns Hopkins University School of Medicine, Baltimore, MD, USA) (Supplementary Material). The cells were cultured in RPMI-1640 cell culture medium supplemented with 10% fetal calf serum, l-glutamine, and antibiotics. Puromycin (2 μg/mL) was used to maintain PSMA expression as previously reported [24]. LNCaP tumor cells (androgen-sensitive, human prostate carcinoma cell line, ACC 256) were obtained from the German Collection of Microorganisms and Cell Cultures (DMSZ) GmbH, Germany (Supplementary Material). The cells were cultured under standard cell culture conditions in RPMI-1640 medium supplemented with 10% fetal calf serum, l-glutamine, antibiotics, and pyruvate (1 mM).

### Uptake and internalization studies

Cell uptake and internalization studies of [^177^Lu]Lu-Ibu-DAB-PSMA and [^177^Lu]Lu-PSMA-617 applied at variable ligand concentrations were performed as previously reported (Supplementary Material) [13]. In brief, PC-3 PIP and LNCaP tumor cells were seeded in 12-well plates (3 × 10^5^ cells and 1 × 10^6^ cells in 2 mL medium/well, respectively) and incubated overnight. Both PSMA radioligands were applied at variable molar concentrations (0.75–500 nM) but at a constant activity concentration of 37.5 kBq/mL. The tumor cells were incubated for 4 h followed by washing steps and detachment of the cells for counting in a γ-counter (Perkin Elmer, Wallac Wizard 1480). The studies were performed in three independent experiments of six replicates. Statistical analysis was performed using a two-way ANOVA with Tukey’s and Sidak’s multiple comparison post-test in Graph Pad Prism (version 8) for different concentrations of the same radioligand and for the two radioligands at the same concentration, respectively. A *p* value of < 0.05 was considered statistically significant.

### In vivo studies

All applicable international, national, and/or institutional guidelines for the care and use of laboratory animals were followed and all animal experiments were carried out according to the guidelines of Swiss Regulations for Animal Welfare. The preclinical studies were ethically approved by the Cantonal Committee of Animal Experimentation and permitted by the responsible cantonal authorities (license No. 75668).

Female and male athymic BALB/c nude mice were obtained from Charles River Laboratories, Germany, at the age of 5–6 weeks. Female mice were subcutaneously inoculated with PSMA-positive PC-3 PIP tumor cells (6 × 10^6^ cells in 100 μL Hanks’ balanced salt solution (HBSS)) on the right shoulder and with PSMA-negative PC-3 flu tumor cells (5 × 10^6^ cells in 100 μL HBSS) on the left shoulder as previously reported [17, 21, 24]. Male BALB/c nude mice were subcutaneously inoculated on the right shoulder with LNCaP tumor cells (5 × 10^6^ cells in PBS mixed with Matrigel (20 mg/mL, BD Biosciences) at a ratio of 3 to 1 (*v/v*) to a total volume of 200 μL per mouse). Biodistribution and SPECT/CT imaging studies with both tumor xenograft models were performed when the tumors reached a volume of 100–300 mm^3^.

### Biodistribution studies

[^177^Lu]Lu-Ibu-DAB-PSMA (5 MBq, 100 µL) and [^177^Lu]Lu-PSMA-617 (5 MBq, 100 µL) were diluted in saline containing 0.05% bovine serum albumin (BSA) to prevent adherence of the radioligands to the syringe. The radioligands were intravenously injected at a ligand amount of 0.05 nmol or 1.0 nmol per mouse. The number of mice per setting was commonly *n* = 3 and, in case of inconclusive results, additional mice up to a total of *n* = 6 were included. The mice were sacrificed at 4 h, 24 h, or 48 h post injection (p.i.) and selected tissues and organs were collected, weighed, and counted in a γ-counter (Supplementary Material). The data sets were analyzed for significance using a one-way ANOVA with Sidak’s multiple comparison post-test using the GraphPad Prism software (version 8). A *p* value of < 0.05 was considered statistically significant. Tumor-to-kidney and tumor-to-blood ratios were calculated for all investigated timepoints after injection of each radioligand.

Time-activity curves over the first 48 h after injection of the radioligands were determined for the PC-3 PIP tumor, the LNCaP tumor, and the kidneys of the corresponding xenograft model based on non-decay-corrected time-dependent biodistribution data obtained at 4 h, 24 h, and 48 h p.i. This allowed calculating the areas under the curve (AUC_0→48 h_) using the GraphPad Prism software (version 8) (Supplementary Material). The tumor-to-kidney AUC_0→48 h_ ratios were calculated for both xenograft models.

### SPECT/CT imaging studies

SPECT/CT experiments were performed using a dedicated small-animal SPECT/CT camera (NanoSPECT/CT™, Mediso Medical Imaging Systems, Budapest, Hungary) as previously reported [17]. The PSMA radioligands were diluted in saline containing 0.05% BSA (8 MBq, 100 µL) and injected at 0.05 nmol or 1.0 nmol per mouse into the lateral tail vein (*n* = 2). SPECT and CT scans were acquired at 1 h, 4 h, and 24 h after injection of the radioligands using the Nucline software (version 1.02, Mediso Ltd., Budapest, Hungary). The real-time CT reconstruction used a cone-beam-filtered backprojection. The reconstruction of SPECT data was performed using the HiSPECT software (version 1.4.3049, Scivis GmbH, Göttingen, Germany). The data were post-processed using VivoQuant (version 3.5, inviCRO Imaging Services and Software, Boston USA). A Gaussian post-reconstruction filter (FWHM = 1.0 mm) was applied and the scale of activity accumulation was set as indicated on the images (minimum value = 0.75 Bq/voxel to maximum value = 15 Bq/voxel).

## Results

### In vitro studies using PC-3 PIP and LNCaP cells

**Fig. 2:**
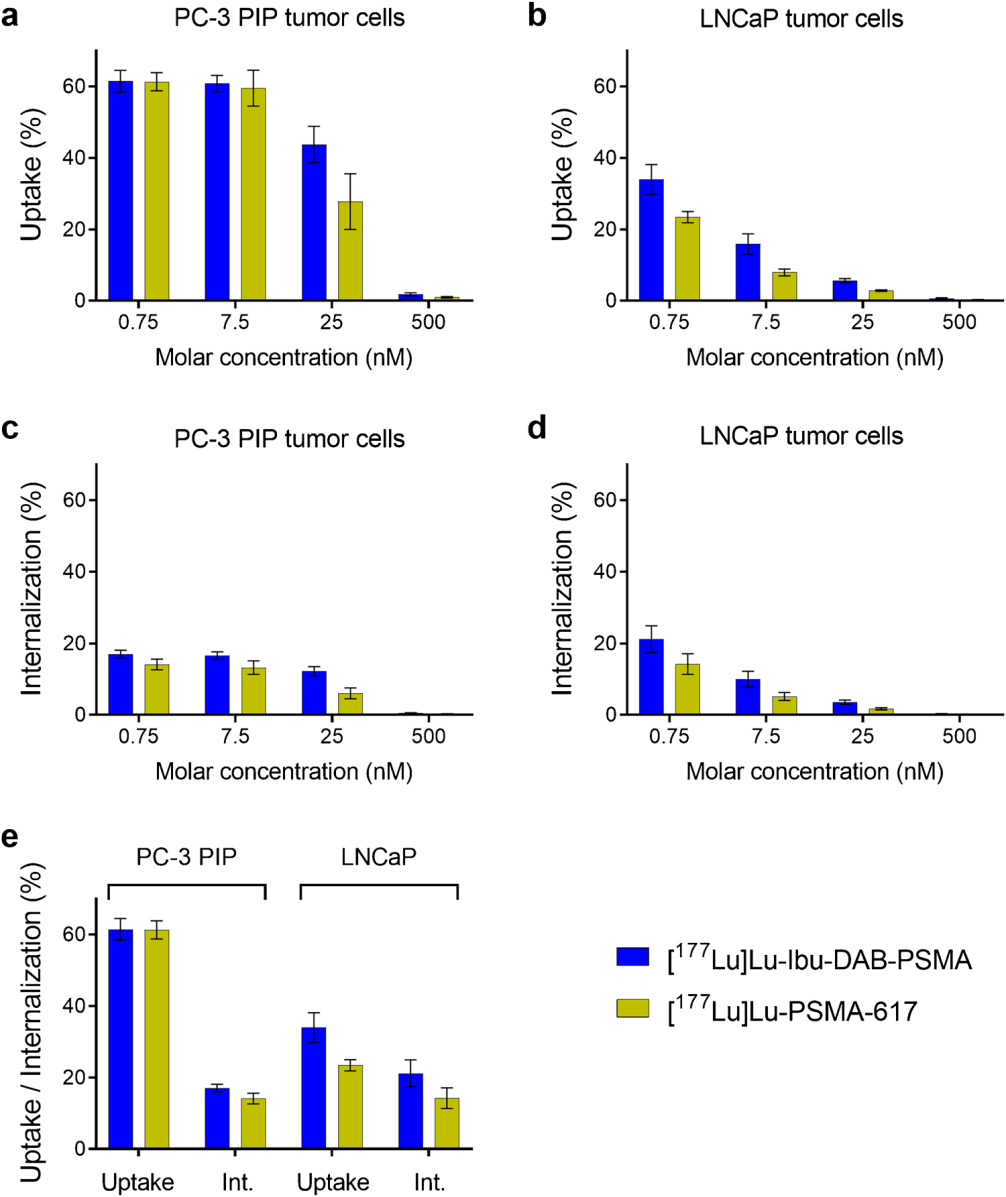
Cell uptake and internalization studies of [^177^Lu]Lu-Ibu-DAB-PSMA and [^177^Lu]Lu-PSMA-617 (average ± SD, *n* = 3). **a/b** Cell uptake in **a** PC-3 PIP and **b** LNCaP tumor cells at variable ligand concentrations. **c/d** Internalization in **c** PC-3 PIP and **d** LNCaP tumor cells at variable ligand concentrations. **e** Comparison of uptake and internalization (Int.) in PC-3 PIP and LNCaP tumor cells at a ligand concentration of 0.75 nM

Cell uptake and internalization of variable concentrations of PSMA ligands were investigated in PC-3 PIP and LNCaP tumor cells (Fig. 2a–d). In PC-3 PIP tumor cells, the uptake and internalization of [^177^Lu]Lu-Ibu-DAB-PSMA and [^177^Lu]Lu-PSMA-617 was equally high (60–61% of total added activity) for the two lowest molar ligand concentrations (0.75 nM and 7.5 nM). At a molar concentration of 25 nM, the uptake was somewhat reduced for both radioligands whereas at 500 nM, the uptake dropped to background levels (≤ 2%) (Fig. 2a). The uptake of both radioligands was up to tenfold lower in LNCaP than in PC-3 PIP tumor cells (Fig. 2a/b). [^177^Lu]Lu-Ibu-DAB-PSMA reached ~ 34% uptake in LNCaP cells at a ligand concentration of 0.75 nM; however, it decreased to ~ 16% at a tenfold higher ligand concentration (7.5 nM; *p* < 0.05) and to ~ 6% at a ligand concentration of 25 nM. [^177^Lu]Lu-PSMA-617 showed the same pattern but significantly (*p* < 0.05) lower uptake of ~ 23% at a ligand concentration of 0.75 nM, ~ 8% at 7.5 nM, and ~ 3% at 25 nM. The uptake of both radioligands was < 1% at a ligand concentration of 500 nM (Fig. 2b). At all ligand concentrations, the internalized fractions followed the same pattern as the total uptake (Fig. 2c/d), but it was much lower for PC-3 PIP tumor cells (23–28% of total uptake) than for LNCaP tumor cells (> 60% of total uptake) (Fig. 2e). The absolute amount of internalized radioligand was, thus, in a similar range (14–21% of total added activity) for both cell lines at a ligand concentration of 0.75 nM.

PSMA-specific uptake of the radioligands in PC-3 PIP tumor cells was indirectly demonstrated by negligible uptake in PSMA-negative PC-3 flu tumor cells (< 1% of total added activity after 4 h incubation). The same observation was made after blocking of PSMA with excess 2-(phosphonomethyl)pentanedioic acid (2-PMPA; 100 µM), which prevented the uptake of radioligands into LNCaP tumor cells (data not shown).

### Tissue distribution profiles in PC-3 PIP and LNCaP tumor mouse models

**Fig. 3:**
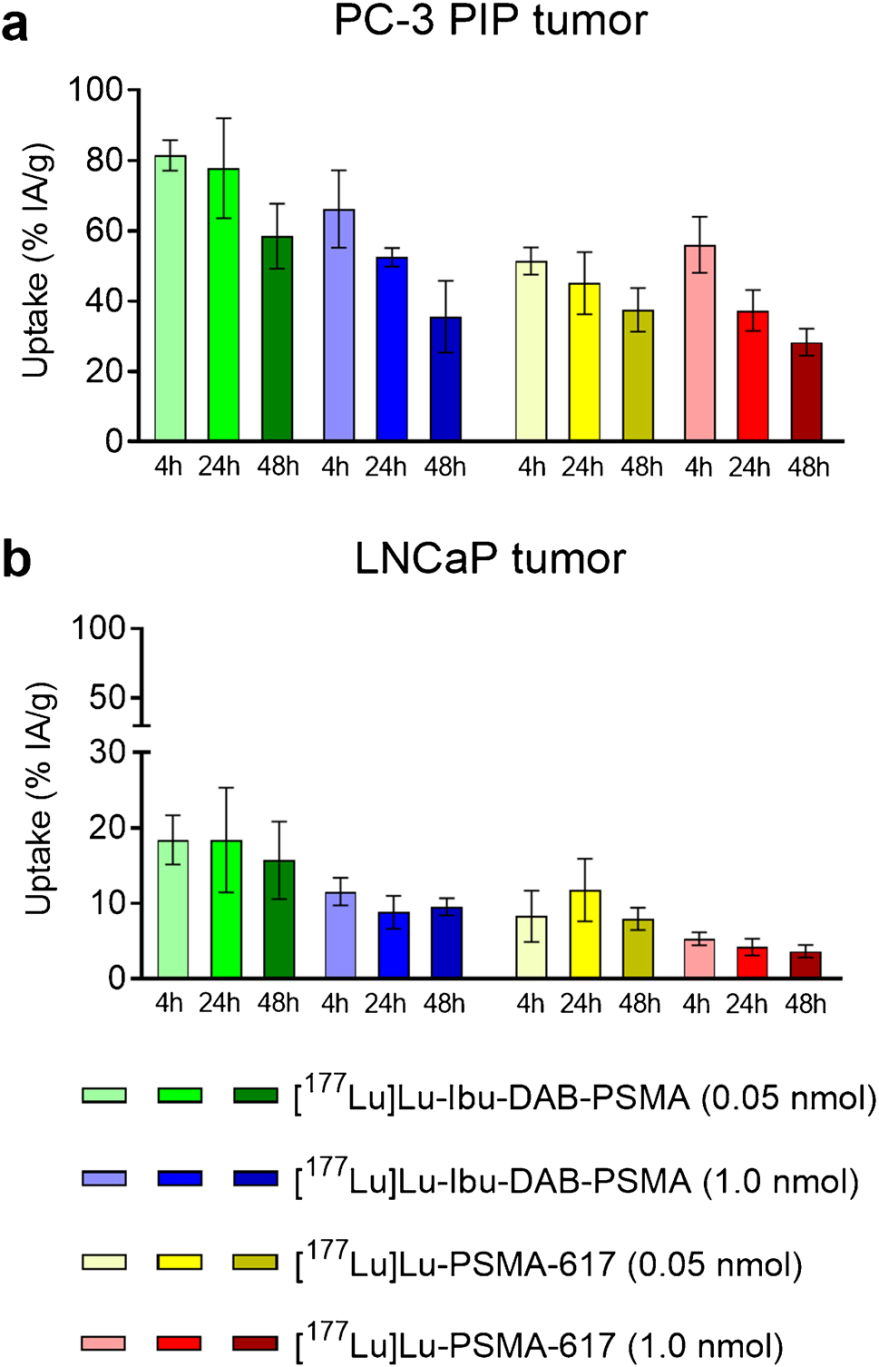
**a/b** Uptake of [^177^Lu]Lu-Ibu-DAB-PSMA and [^177^Lu]Lu-PSMA-617 in **a** PC-3 PIP tumors and **b** LNCaP tumors using a ligand amount of 0.05 nmol and 1.0 nmol per mouse.

Biodistribution studies were performed in PC-3 PIP and LNCaP tumor mouse models injected with low and high molar ligand amounts (Fig. 3; Table 1; Supplementary Material, Tables S1–S4). Comparison of low and high molar ligand amounts revealed that the PC-3 PIP tumor uptake of [^177^Lu]Lu-Ibu-DAB-PSMA was slightly increased after injection of a low ligand amount at 4 h p.i. (*p* > 0.05) but significantly higher (1.5–1.6-fold) at 24 h and 48 h p.i. (*p* < 0.05) (Fig. 3a). The uptake of [^177^Lu]Lu-PSMA-617 in PC-3 PIP tumors was almost constant at all timepoints irrespective of the injected molar amount of ligand (*p* > 0.05) (Fig. 3a). In the LNCaP tumor mouse model, the tumor uptake was about 1.5–threefold higher for both radioligands when applied at the low molar amount (0.05 nmol per mouse) compared to the results after injection of a high molar amount (1.0 nmol per mouse) irrespective of the investigated timepoint (Fig. 3b).

**Table 1:** Tissue distribution data in PC-3 PIP/flu or LNCaP tumor-bearing mice obtained at 4 h, 24 h, and 48 h after injection of [^177^Lu]Lu-Ibu-DAB-PSMA or [^177^Lu]Lu-PSMA-617 injected at either 0.05 or 1.0 nmol ligand amount. The values are indicated as average ± SD obtained from each group of mice (*n* = 3 − 6) and listed as percentage of injected activity per gram tissue [% IA/g]

[^177^Lu]Lu-Ibu-DAB-PSMA						
Time	4 h p.i	4 h p.i.	24 h p.i	24 h p.i.	48 h p.i	48 h p.i.
Ligand amount	0.05 nmol	1.0 nmol^a^	0.05 nmol	1.0 nmol^a^	0.05 nmol	1.0 nmol^a^
PC-3 PIP model						
Blood	1.3 ± 0.3	3.7 ± 0.5	0.23 ± 0.03	0.16 ± 0.02	0.13 ± 0.03	0.10 ± 0.04
Kidneys	69 ± 10	19 ± 2	5.5 ± 1.0	6.0 ± 0.7	2.3 ± 0.4	4.1 ± 0.8
PC-3 PIP tumor^c^	81 ± 4	66 ± 11	78 ± 14	52 ± 3	58 ± 9	36 ± 10
LNCaP model						
Blood	1.6 ± 0.3	0.87 ± 0.29	0.16 ± 0.03	0.19 ± 0.03	0.10 ± 0.02	0.09 ± 0.02
Kidneys	58 ± 10	16 ± 1	5.9 ± 1.5	6.8 ± 1.1	2.0 ± 0.3	2.4 ± 0.5
LNCaP tumor	18 ± 3	12 ± 2	18 ± 7	8.8 ± 2.2	16 ± 5	9.5 ± 1.1
[^177^Lu]Lu-PSMA-617						
Time	4 h p.i	4 h p.i.	24 h p.i.	24 h p.i.	48 h p.i.	48 h p.i.
Ligand amount	0.05 nmol	1.0 nmol^b^	0.05 nmol	1.0 nmol^b^	0.05 nmol	1.0 nmol^b^
PC-3 PIP model						
Blood	0.06 ± 0.01	< 0.05	< 0.05	< 0.05	< 0.05	< 0.05
Kidneys	13 ± 5	3.7 ± 1.1	1.4 ± 0.5	0.76 ± 0.15	0.86 ± 0.15	0.35 ± 0.05
PC-3 PIP tumor^c^	51 ± 4	56 ± 8	45 ± 9	37 ± 6	37 ± 6	28 ± 4
LNCaP model						
Blood	< 0.05	< 0.05	< 0.05	< 0.05	< 0.05	< 0.05
Kidneys	14 ± 9	2.9 ± 0.6	0.86 ± 0.13	0.67 ± 0.18	0.46 ± 0.06	0.31 ± 0.10
LNCaP tumor	8.3 ± 3.4	5.3 ± 0.9	12 ± 4	4.2 ± 1.1	7.9 ± 1.5	3.7 ± 0.8

^a^Data obtained with 1.0 nmol [^177^Lu]Lu-Ibu-DAB-PSMA in the PC-3 PIP mouse model were reproduced from Deberle et al. [21]

^b^Data obtained with 1.0 nmol [^177^Lu]Lu-PSMA-617 in the PC-3 PIP mouse model were reproduced from Benešová et al. [13]

^c^Data referring to the PC-3 flu tumor are reported in the Supplementary Material

Comparison of the absolute tumor uptake after injection of 0.05 nmol [^177^Lu]Lu-Ibu-DAB-PSMA or [^177^Lu]Lu-PSMA-617 revealed an approximately fourfold higher accumulation in PC-3 PIP tumor xenografts (78 ± 14% IA/g and 45 ± 9% IA/g at 24 h p.i.) than in LNCaP tumor xenografts (18 ± 7% IA/g and 12 ± 4% IA/g at 24 h p.i.). Injection of a higher molar amount of these ligands (1.0 nmol per mouse) resulted in even larger differences between the mouse models. The tumor uptake was 6- and ninefold higher in PC-3 PIP tumor xenografts (52 ± 3% IA/g and 37 ± 6% IA/g at 24 h p.i.) than in LNCaP tumor xenografts (8.8 ± 2.2% IA/g and 4.2 ± 1.1% IA/g at 24 h p.i.). This substantial difference in activity accumulation between the xenograft types was observed at all investigated timepoints and can be ascribed to the significantly higher PSMA expression level in PC-3 PIP tumors than in LNCaP tumors (Fig. 3a/b).

The uptake of activity in the kidneys was particularly affected at early timepoints when varying the amount of injected PSMA ligand. At 4 h p.i., the kidney retention of [^177^Lu]Lu-Ibu-DAB-PSMA and [^177^Lu]Lu-PSMA-617 was about fourfold increased at low amount of injected ligand compared to a high amount (58–69% IA/g vs. 16–19% IA/g; *p* < 0.05 and 13–14% IA/g vs. 2.9–3.7% IA/g *p* < 0.05, respectively). At later time points, renal retention of activity was in the same range (5.5–6.8% IA/g, 24 h p.i.) irrespective of the injected ligand amount of [^177^Lu]Lu-Ibu-DAB-PSMA. The same held true for the kidney retention of [^177^Lu]Lu-PSMA-617, which was, however, in a considerably lower range (0.67–1.4% IA/g, 24 h p.i.) (Table 1).

The activity retention in other organs and tissues including the blood, liver, bone, and salivary glands was not significantly affected by the amount of injected ligand for both [^177^Lu]Lu-Ibu-DAB-PSMA and [^177^Lu]Lu-PSMA-617 (Table 1; Supplementary Material, Table S1-S4).

### Tumor-to-background ratios resulting from variable preclinical settings

**Fig. 4:**
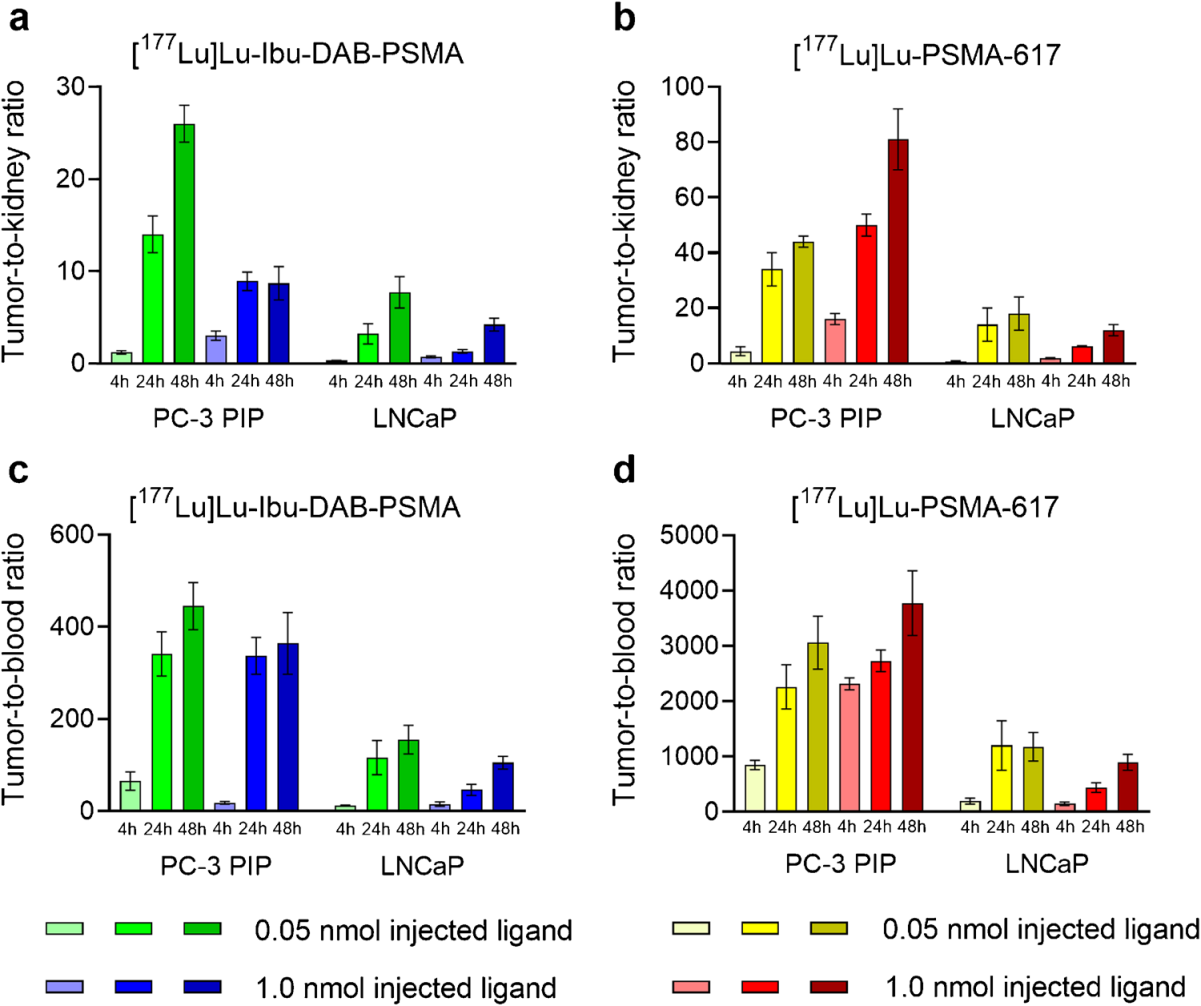
Tumor-to-background ratios based on biodistribution data obtained in PC-3 PIP and LNCaP tumor-bearing female and male mice, respectively. **a** Tumor-to-kidney ratios of [^177^Lu]Lu-Ibu-DAB-PSMA. **b** Tumor-to-kidney ratios of [^177^Lu]Lu-PSMA-617. **c** Tumor-to-blood ratios of [^177^Lu]Lu-Ibu-DAB-PSMA. **d** Tumor-to-blood ratios of [^177^Lu]Lu-PSMA-617. The values represent the average ± SD of values obtained from *n* = 3 − 6 mice. Data obtained with 1.0 nmol PSMA-617 and Ibu-DAB-PSMA in the PC-3 PIP xenograft model were previously published by Benešová et al. [13] and Deberle et al. [21]

As a result of the changes in activity accumulation in response to the tumor model and injected ligand amount, the tumor-to-background ratios varied among the various settings. The tumor-to-kidney ratios 4 h after injection of [^177^Lu]Lu-Ibu-DAB-PSMA were somewhat lower when using 0.05 nmol compared to 1.0 nmol (1.2 ± 0.2 vs. 3.0 ± 0.5, *p* > 0.05 and 0.32 ± 0.03 vs. 0.71 ± 0.10; *p* > 0.05) for the PC-3 PIP and LNCaP tumor mouse model, respectively. At the 24 h p.i. timepoint, these ratios were, however, significantly higher after application of 0.05 nmol radioligand compared to 1.0 nmol (14 ± 2 vs. 8.9 ± 1.0, *p* < 0.05 and 3.2 ± 1.1 vs. 1.3 ± 0.2; *p* < 0.05) in PC-3 PIP and LNCaP tumor-bearing mice with a similar result also obtained at 48 h p.i. (Fig. 4a). At all investigated timepoints, the tumor-to-kidney ratios of [^177^Lu]Lu-PSMA-617 in PC-3 PIP tumor-bearing mice were, however, significantly increased when using 1.0 nmol ligand instead of 0.05 nmol (*p* < 0.05) (Fig. 4b). In contrast, the ratios of [^177^Lu]Lu-PSMA-617 in the LNCaP tumor mouse model showed an opposite trend with higher tumor-to-kidney ratios after injection of low amounts of ligand.

The tumor-to-blood ratios were increased at low molar amounts of injected ligand in the PC-3 PIP mouse model when using [^177^Lu]Lu-Ibu-DAB-PSMA, but an opposite trend was observed for [^177^Lu]Lu-PSMA-617 (Fig. 4c/d). In the LNCaP tumor mouse model, the tumor-to-blood ratios were higher when using low amounts of ligand irrespective of whether [^177^Lu]Lu-Ibu-DAB-PSMA or [^177^Lu]Lu-PSMA-617 was used (Fig. 4c/d).

Calculation of the areas under the curve over the first 48 h after application of the radioligands (AUC_0→48 h_) and the respective ratios revealed consistently increased tumor-to-kidney ratios for high molar amounts of injected ligand. In the case of [^177^Lu]Lu-Ibu-DAB-PSMA, this AUC_0→48 h_ ratio was 1.8-fold higher in the PC-3 PIP mouse model and 1.3-fold in the LNCaP. In the case of [^177^Lu]Lu-PSMA-617, the ratios were 2.8-fold increased in the PC-3 PIP model and 1.6-fold in the LNCaP model. (Table 2; Supplementary Material, Table S5).

**Table 2:** Tumor-to-kidney ratios of the areas under the curve over the first 48 h (AUC_Tu(0→48 h)_-to-AUC_Ki(0→48 h)_) indicated as average ± SE. The AUC_0→48 h_ values were based on non-decay-corrected biodistribution data obtained at 4 h, 24 h, and 48 h after injection of either 0.05 or 1.0 nmol of [^177^Lu]Lu-Ibu-DAB-PSMA or [^177^Lu]Lu-PSMA-617 in either PC-3 PIP/flu or LNCaP tumor-bearing mice

Radioligand	[^177^Lu]Lu-Ibu-DAB-PSMA	[^177^Lu]Lu-Ibu-DAB-PSMA	[^177^Lu]Lu-PSMA-617	[^177^Lu]Lu-PSMA-617
Ligand amount	0.05 nmol	1.0 nmol	0.05 nmol	1.0 nmol
PC-3 PIP model^a^: AUC_Tu_-to-AUC_Ki_	3.2 ± 0.5	5.6 ± 0.7	9.8 ± 3.6	27 ± 7
LNCaP model^b^: AUC_Tu_-to-AUC_Ki_	0.89 ± 0.25	1.2 ± 0.2	2.2 ± 1.5	3.5 ± 0.9

^a^PC-3 PIP tumors were grown in female mice; ^b^LNCaP tumors were grown in male mice

### SPECT/CT imaging studies

**Fig. 5:**
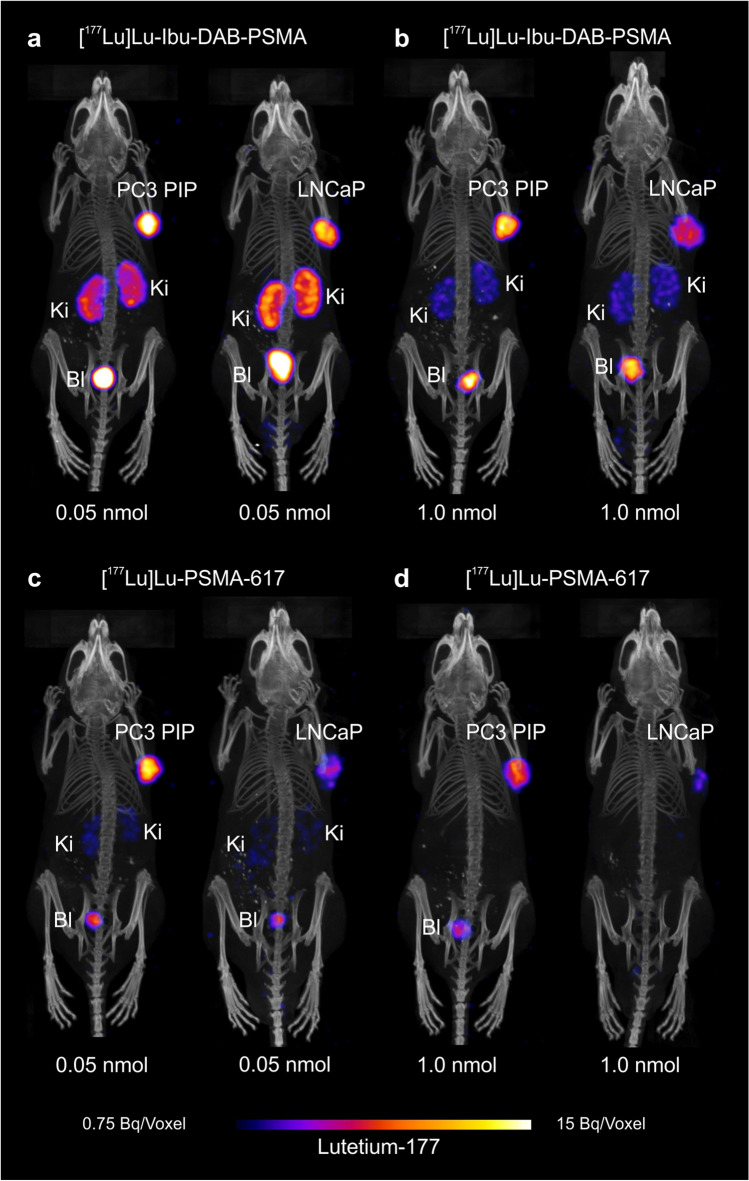
SPECT/CT images as maximum intensity projections (MIPs) of PC-3 PIP and LNCaP tumor-bearing mice at 4 h after injection of either 0.05 nmol or 1.0 nmol of [^177^Lu]Lu-Ibu-DAB-PSMA or [^177^Lu]Lu-PSMA-617. **a/b** Images of mice after injection of **a** 0.05 nmol or **b** 1.0 nmol [^177^Lu]Lu-Ibu-DAB-PSMA (8 MBq). **c/d** Images of mice after injection of **c** 0.05 nmol or **d** 1.0 nmol [^177^Lu]Lu-PSMA-617 (8 MBq). PC-3 PIP = PSMA-positive PC-3 PIP tumor; LNCaP = PSMA-positive LNCaP tumor; Ki = kidney; Bl = urinary bladder

SPECT/CT imaging studies demonstrated the generally higher accumulation of both radioligands in the PC-3 PIP tumors than in the LNCaP tumor xenografts. The images acquired within the first 4 h after injection of [^177^Lu]Lu-Ibu-DAB-PSMA and [^177^Lu]Lu-PSMA-617, respectively, confirmed the findings of the biodistribution study in which the uptake in tumors and in the kidneys was increased when 0.05 nmol ligand was applied compared to 1.0 nmol (Fig. 5, Supplementary Material, Fig. S1/S2). After 24 h, the tumor uptake of both radioligands was still higher after injection of 0.05 nmol than after injection of 1.0 nmol ligand. At this timepoint, the activity was, however, almost entirely cleared from the kidneys, irrespective of the radioligand, the tumor mouse model, and the amount of injected ligand (Supplementary Material, Fig. S1/S2).

## Discussion

This study demonstrated the relevance of the chosen tumor model and the molar amount of applied ligand with regard to the resultant cell uptake and tissue distribution profile of PSMA radioligands.

The in vitro investigations using PC-3 PIP and LNCaP tumor cells demonstrated that the molar concentration of applied radioligand critically affected the results. The considerably higher PSMA expression level in PC-3 PIP cells compared to LNCaP cells [16, 17] makes PC-3 PIP cells less sensitive to saturation effects. It is, thus, advisable to use low radioligand concentrations (< 0.75 nM) for experiments with LNCaP cells, as the maximum uptake would otherwise be limited by the PSMA expression level rather than reflect the differences among the radioligands in question. An additional interesting finding of this study was that the internalization was considerably more efficient in LNCaP cells (> 60% of total uptake) than in PC-3 PIP cells (~ 25% of total uptake). The reason for this observation remains unknown and was not further investigated in this study. It can be speculated, however, that the higher internalization rate of LNCaP tumor cells is a result of a potential function of PSMA in LNCaP tumor cells, which is not the case for the artificial PSMA-expressing PC-3 PIP cell line.

In line with the in vitro results, the biodistribution data demonstrated that LNCaP tumors are particularly susceptible to the change of injected molar amount of radioligand due to saturation effects. As a result, the activity accumulation in LNCaP tumors was considerably higher when a low molar amount of radioligand was injected. In PC-3 PIP tumors, saturation effects were less likely to happen; hence, the increase in uptake after injection of a low molar ligand amount was only moderate. The absolute tumor uptake of the radioligands was, however, higher in PC-3 PIP tumors than in LNCaP Tumors, which is in line with recent findings of Current et al., who demonstrated that the uptake of [^177^Lu]Lu-PSMA-617 correlates with the PSMA expression level [25].

While other studies investigated the effect of variable amounts of injected ligand on the tumor uptake of conventional PSMA radioligands [26–29], this study showed for the first time how the molar ligand amount affected the uptake of an albumin-binding radioligand. It was demonstrated that in tumors that express PSMA at low levels such as LNCaP, the amount of injected ligand was relevant for the uptake of both the conventional and the albumin-binding radioligand. In contrast, the uptake in PC-3 PIP xenografts, which express PSMA at high levels, is more affected by the injected ligand amount in the case of the albumin-binding [^177^Lu]Lu-Ibu-DAB-PSMA than in the case of [^177^Lu]Lu-PSMA-617.

Although the present study demonstrated that a low molar amount of injected radioligand was favorable to achieve high tumor accumulation, it also showed that it affected the kidney uptake unfavorably. This resulted in reduced tumor-to-kidney ratios in particular at early timepoints as visualized on SPECT/CT images acquired 1 h after injection of the radioligands (Supplemenatry Material, Fig. S1 and Fig. S2). Calculation of the tumor-to-kidney AUC ratios over the first 48 h revealed that high molar amounts of injected ligand would provide a potentially improved safety profile for the kidneys. Whether the first 48 h are decisive for the tumor-to-kidney dose ratio may, however, be questioned, given the fact that PSMA radioligands are effectively cleared via kidneys over time while the activity accumulated in the tumor is well retained over time.

Conclusions about potential differences in blood retention of activity after injection of low and high molar amounts of ligands are not feasible as the blood values were already low 4 h after injection of the radioligands. As previously reported, our study confirmed, however, that [^177^Lu]Lu-Ibu-DAB-PSMA was more retained in the blood than [^177^Lu]Lu-PSMA-617 due to its albumin-binding properties [21]. As the LNCaP tumor uptake of both radioligands was higher after injection of the low molar ligand amount, the tumor-to-blood ratios were also increased in this tumor mouse model. The same held true for [^177^Lu]Lu-Ibu-DAB-PSMA in PC-3 PIP tumor-bearing mice; however, in this setting, [^177^Lu]Lu-PSMA-617 showed more favorable tumor-to-blood ratios early after injection of the high molar ligand amount.

From a practical perspective, it is important to mention that the reproducible tumor take and predictable growth of the PC-3 PIP tumors are favorable characteristics of a tumor mouse model for the planning of preclinical studies. LNCaP cells, on the other hand, are commonly applied with Matrigel to ensure tumor development in vivo and the tumor take and growth varies substantially among individual mice. This situation complicates the use of the LNCaP tumor mouse model to screen radioligands but also in view of preclinical therapy studies.

Finally, it is also important to note that the amount of injected ligand should be the same for the evaluation of biodistribution and therapy studies even if the applied activity may be different. Only under such conditions, dosimetry estimation relevant to the therapeutic setting can be based on biodistribution data. The smallest applicable molar amount of injected ligand would, thus, also be dependent on the labeling capacity of the respective radionuclide to allow injecting therapeutic quantities of activity.

Overall, the impact of the parameters investigated in this study warrants a critical consideration of the current practice in preclinical research, but may also be relevant in clinical settings. The expression levels of PSMA in patients may vary depending on the stage of the disease [30–32] and the molar amount of injected radioligand may affect the distribution profile as demonstrated in silico [33, 34]. This is of relevance with respect to the absorbed tumor dose but also regarding undesired side effects to normal tissue.

## Conclusion

This work demonstrated the impact of the molar amount of injected ligand and the tumor mouse model, respectively, on the resultant data of preclinically evaluated radioligands. In this particular study, low amounts of injected ligand resulted in a more favorable tumor uptake than high amounts. The opposite was true, however, for the kidney uptake and, hence, unfavorably low tumor-to-kidney ratios were obtained at early time points after injection of a low molar ligand amount. These findings emphasize the importance of defining the molar amount of injected ligand—in addition to the injected activity—in order to enable comparison of different radioligands. More consistent protocols would, thus, be desirable in preclinical but possibly also in clinical settings. It may enable better comparison of therapy responses of patients treated in different hospitals and possibly facilitate the optimization of application schemes.

## Supplementary Information

Supplementary Material comprises information about the employed cell lines, in vitro experiments, biodistribution studies including AUC values and SPECT/CT imaging results.

Supplementary file 1 (PDF 2.18 mb)

## Abbreviations

ANOVA: Analysis of variance
AUC: Area under the curve
Bl: Urinary bladder
BSA: Bovine serum albumin
CT: Computed tomography
DAB: Diaminobutyric acid
FWHM: Full width at half maximum
HBSS: Hanks’ balanced salt solution
HPLC: High performance liquid chromatography
IA: Injected activity
Ki: Kidney
mCRPC: Metastatic castration-resistant prostate cancer
MIP: Maximum intensity projection
PBS: Phosphate-buffered saline
p.i.: Post injection
2-PMPA: 2-(Phosphonomethyl) pentanedioic acid
PSMA: Prostate-specific membrane antigen
SD: Standard deviation
SE: Standard error
SPECT: Single photon emission computed tomography
Tu: Tumor
