# Supplementary material for: Impact of the mouse model and molar amount of injected ligand on the tissue distribution profile of PSMA radioligands
Source: Eur J Nucl Med Mol Imaging. 2021 Aug 17;49(2):470–80. doi: 10.1007/s00259-021-05446-5 (PMC8803738; doi:10.1007/s00259-021-05446-5)
Supplement: Supplementary file 1 — (PDF 2.18 mb) [file 259_2021_5446_MOESM1_ESM.pdf]

Supplementary Material

Impact of the mouse model and molar amount of injected ligand on the tissue distribution profile of PSMA radioligands

Viviane J. Tschan[1], Francesca Borgna[1], Roger Schibli[1,2], Cristina Müller[1,2*]

1. Center for Radiopharmaceutical Sciences ETH-PSI-USZ, Paul Scherrer Institute, 5232 Villigen-PSI, Switzerland
2. Department of Chemistry and Applied Biosciences, ETH Zurich, 8093 Zurich, Switzerland

E-Mail addresses:
viviane.tschan@psi.ch; francesca.borgna@psi.ch; roger.schibli@psi.ch; cristina.mueller@psi.ch

***Correspondence to:**
PD Dr. Cristina Müller
Center for Radiopharmaceutical Sciences ETH-PSI-USZ
Paul Scherrer Institute
5232 Villigen-PSI
Switzerland
e-mail: cristina.mueller@psi.ch
phone: +41-56-310 44 54; fax: +41-56-310 28 49

1. Cell lines

PC-3 PIP/flu cells: Sublines of the androgen-independent PC-3 human prostate cancer cell line originally derived from an advanced androgen-independent bone metastasis. The sublines were previously created by transduction to express PSMA at high levels (PSMA-positive PC-3 PIP cells) or by mock transduction to obtain a cell line which does not express PSMA (PSMA-negative PC-3 flu cells) [1-3]. Both cell lines have been used by different research groups including our own group to evaluate PSMA-targeting radioligands [4-7].

LNCaP cells: LNCaP cells are androgen-sensitive prostate cancer cells, originally derived from a metastatic lesion of human prostatic adenocarcinoma, which expresses PSMA naturally [8]. LNCaP tumor cells and the respective tumor mouse model were previously used to evaluate PSMA-targeting radioligands [9-14].

Both tumor cell types were kept in a humidified atmosphere at 37 °C and 5% CO_2. Routine cell culture was performed twice a week using PBS/EDTA (2 mM) and trypsin for cell detachment.

2. Cell uptake and internalization studies

Purpose: The goal was to determine the impact of the molar ligand concentration on the uptake and internalization of the radioligands into PC-3 PIP and LNCaP tumor cells.

Methods: Uptake and internalization studies were performed with PC-3 PIP and LNCaP tumor cells using variable concentrations of the respective ligand but constant activity concentrations. PC-3 PIP and LNCaP cells were seeded in 12-well plates and incubated at 37 °C and 5% CO_2 overnight. Experiments with LNCaP cells were performed with poly-L-lysine-coated well plates to ensure better cell adherence. The radioligand solution was diluted using saline containing 0.05% bovine serum albumin (BSA) to avoid adherence to the vial. A defined volume of 25 µL (corresponding to 37.5 kBq) of this solution was added to the cell monolayers after the addition of 975 µL cell culture medium without supplements. The final molar concentrations in each well ranged from 0.75 nM to 500 nM. After incubation of the cells for 4 h, several washing steps were performed using PBS for determination of the total uptake. A glycine buffer (pH 2.8) was used to strip cells in order to determine the internalized fraction of the radioligands. The cells were lysed with NaOH (1 M) and the activity counted using a γ-counter (Perkin Elmer, Wallac Wizard 1480). The protein content in each well was determined using a commercial kit (Micro BCA Protein Assay kit; Pierce, Thermo Scientific) in order to standardize the measured activity to the amount of proteins in each well. Each experimental setting was tested in three independent experiments performed in six replicates with PC-3 PIP and LNCaP tumor cells, respectively.

Results: The results are reported in the main article.

3. Biodistribution studies

Purpose: The impact of the tumor mouse model and the injected ligand amount on the biodistribution of [^{177}Lu]Lu-Ibu-DAB-PSMA and [^{177}Lu]Lu-PSMA-617 was investigated.

Methods: The biodistribution studies were conducted with either PC-3 PIP/flu or LNCaP tumor-bearing mice injected with either 0.05 nmol or 1.0 nmol of radioligand per mouse, whereas the applied activity was kept constant for each setting. The results were decay-corrected and the values indicated as average ± SD obtained from each group of mice (n = 3−6) and listed as percentage of injected activity per gram tissue [% IA/g].

Results: The results are listed below (Table S1–S4) and described and discussed in the main article.

Table S1 Decay-corrected biodistribution data of [^{177}Lu]Lu-Ibu-DAB-PSMA in PC-3 PIP/flu tumor-bearing mice injected with either 0.05 nmol or 1.0 nmol per mouse. The values are indicated as average ± SD obtained from each group of mice (n = 3−6) and listed as percentage of injected activity per gram tissue [% IA/g].

[^{177}Lu]Lu-Ibu-DAB-PSMA						
**Dissection time**	**4 h p.i.**		**24 h p.i.**		**48 h p.i.**	
**nmol per mouse**	**0.05 nmol**	**1.0 nmol[a]**	**0.05 nmol**	**1.0 nmol[a]**	**0.05 nmol**	**1.0 nmol[a]**
Blood	1.3 ± 0.3	3.7 ± 0.5	0.23 ± 0.03	0.16 ± 0.02	0.13 ± 0.03	0.10 ± 0.04
Heart	0.54 ± 0.12	1.3 ± 0.1	0.15 ± 0.02	0.10 ± 0.01	<0.10	<0.10
Lung	1.3 ± 0.3	2.4 ± 0.3	0.27 ± 0.04	0.21 ± 0.03	0.18 ± 0.04	0.14 ± 0.04
Spleen	1.9 ± 0.4	1.1 ± 0.1	0.42 ± 0.09	0.32 ± 0.03	0.25 ± 0.05	0.26 ± 0.08
Kidneys	69 ± 10	19 ± 2	5.5 ± 1.0	6.0 ± 0.7	2.3 ± 0.4	4.1 ± 0.8
Stomach	0.33 ± 0.09	0.62 ± 0.12	0.11 ± 0.02	0.18 ± 0.13	0.12 ± 0.08	<0.10
Intestines	0.29 ± 0.05	0.71 ± 0.05	0.11 ± 0.03	0.11 ± 0.03	<0.10	<0.10
Liver	1.1 ± 0.1	1.5 ± 0.1	0.54 ± 0.13	0.56 ± 0.10	0.28 ± 0.03	0.49 ± 0.08
Muscle	0.22 ± 0.06	0.54 ± 0.09	<0.10	0.05 ± 0.01	<0.10	<0.10
Bone	0.29 ± 0.06	0.62 ± 0.08	0.13 ± 0.04	<0.10	<0.10	<0.10
Salivary glands	0.69 ± 0.18	0.86 ± 0.38	0.21 ± 0.02	0.56 ± 0.37	0.11 ± 0.02	<0.10
PC-3 PIP tumor	81 ± 4	66 ± 11	78 ± 14	52 ± 3	58 ± 9	36 ± 10
PC-3 flu tumor	0.67 ± 0.10	1.2 ± 0.3	0.31 ± 0.05	0.17 ± 0.01	0.17 ± 0.01	0.15 ± 0.04
Tumor-to-blood	65 ± 20	18 ± 3	341 ± 48	337 ± 40	445 ± 51	364 ± 67
Tumor-to-liver	74 ± 8	45 ± 8	149 ± 34	98 ± 21	205 ± 10	72 ± 11
Tumor-to-kidney	1.2 ± 0.2	3.0 ± 0.5	14 ± 2	8.9 ± 1.0	26 ± 2	8.7 ± 1.8

[a] Data obtained with 1.0 nmol [^{177}Lu]Lu-Ibu-DAB-PSMA were reproduced from Deberle et al. [7]

Table S2 Decay-corrected biodistribution data of [^{177}Lu]Lu-PSMA-617 in PC-3 PIP/flu tumor-bearing mice injected with either 0.05 nmol or 1.0 nmol per mouse. The values are indicated as average ± SD obtained from each group of mice (n = 3−6) and listed as percentage of injected activity per gram tissue [% IA/g].

[^{177}Lu]Lu-PSMA-617						
	**4 h p.i.**		**24 h p.i.**		**48 h p.i.**	
	**0.05 nmol**	**1.0 nmol[a]**	**0.05 nmol**	**1.0 nmol[a]**	**0.05 nmol**	**1.0 nmol[a]**
Blood	<0.10	<0.10	<0.10	<0.10	<0.10	<0.10
Heart	<0.10	<0.10	<0.10	<0.10	<0.10	<0.10
Lung	0.12 ± 0.02	<0.10	<0.10	<0.10	<0.10	<0.10
Spleen	0.31 ± 0.16	0.15 ± 0.31	<0.10	<0.10	<0.10	<0.10
Kidneys	13 ± 5	3.7 ± 1.1	1.4 ± 0.5	0.76 ± 0.15	0.86 ± 0.15	0.35 ± 0.05
Stomach	0.31 ± 0.22	<0.10	<0.10	<0.10	<0.10	<0.10
Intestines	1.2 ± 1.9	<0.10	<0.10	<0.10	<0.10	<0.10
Liver	0.15 ± 0.06	<0.10	<0.10	<0.10	<0.10	<0.10
Muscle	<0.10	<0.10	<0.10	<0.10	<0.10	<0.10
Bone	<0.10	<0.10	<0.10	<0.10	<0.10	<0.10
Salivary glands	<0.10	<0.10	<0.10	<0.10	<0.10	<0.10
PC-3 PIP tumor	51 ± 4	56 ± 8	45 ± 9	37 ± 6	37 ± 6	28 ± 4
PC-3 flu tumor	<0.10	<0.10	<0.10	<0.10	<0.10	<0.10
Tumor-to-blood	849 ± 84	2315 ± 108	2262 ± 400	2730 ± 195	>3000	>3000
Tumor-to-liver	387 ± 131	598 ± 108	565 ± 47	528 ± 51	523 ± 32	710 ± 97
Tumor-to-kidney	4.4 ± 1.6	16 ± 2	34 ± 6	50 ± 4	44 ± 2	81 ± 11

[a] Data obtained with 1.0 nmol [^{177}Lu]Lu-PSMA-617 were reproduced from Benesova et al. [15]

Table S3 Decay-corrected biodistribution data of [^{177}Lu]Lu-Ibu-DAB-PSMA in LNCaP tumor-bearing mice injected with either 0.05 nmol or 1.0 nmol per mouse. The values are indicated as average ± SD obtained from each group of mice (n = 3−6) and listed as percentage of injected activity per gram tissue [% IA/g].

[^{177}Lu]Lu-Ibu-DAB-PSMA						
	**4 h p.i.**		**24 h p.i.**		**48 h p.i.**	
	**0.05 nmol**	**1.0 nmol**	**0.05 nmol**	**1.0 nmol**	**0.05 nmol**	**1.0 nmol**
Blood	1.6 ± 0.3	0.87 ± 0.29	0.16 ± 0.03	0.19 ± 0.03	0.10 ± 0.02	<0.10
Heart	0.61 ± 0.13	0.41 ± 0.11	0.11 ± 0.02	0.12 ± 0.02	<0.10	<0.10
Lung	1.4 ± 0.2	0.78 ± 0.19	0.24 ± 0.03	0.23 ± 0.03	0.16 ± 0.04	0.14 ± 0.03
Spleen	1.5 ± 0.4	0.45 ± 0.08	0.35 ± 0.06	0.21 ± 0.06	0.31 ± 0.08	0.24 ± 0.04
Kidneys	58 ± 10	16 ± 1	5.9 ± 1.5	6.8 ± 1.1	2.0 ± 0.3	2.4 ± 0.5
Stomach	0.32 ± 0.06	0.22 ± 0.06	0.13 ± 0.04	0.23 ± 0.10	<0.10	<0.10
Intestines	0.33 ± 0.03	0.23 ± 0.05	0.10 ± 0.01	0.12 ± 0.03	<0.10	<0.10
Liver	1.3 ± 0.1	0.82 ± 0.09	0.48 ± 0.08	0.45 ± 0.07	0.50 ± 0.10	0.57 ± 0.09
Muscle	1.5 ± 1.9	0.22 ± 0.14	<0.10	<0.10	<0.10	<0.10
Bone	0.63 ± 0.33	0.39 ± 0.17	<0.10	0.14 ± 0.03	<0.10	<0.10
Salivary glands	0.59 ± 0.14	0.38 ± 0.08	0.18 ± 0.03	0.16 ± 0.02	0.11 ± 0.01	<0.10
LNCaP tumor	18 ± 3	12 ± 2	18 ± 7	8.8 ± 2.2	16 ± 5	9.5 ± 1.1
Tumor-to-blood	12 ± 1	15 ± 5	116 ± 37	46 ± 12	155 ± 31	105 ± 14
Tumor-to-liver	14 ± 1	14 ± 1	39 ± 13	20 ± 3	32 ± 7	17 ± 2
Tumor-to-kidney	0.32 ± 0.03	0.71 ± 0.10	3.2 ± 1.1	1.3 ± 0.2	7.7 ± 1.7	4.2 ± 0.7

Table S4 Decay-corrected biodistribution data of [^{177}Lu]Lu-PSMA-617 in LNCaP tumor-bearing mice injected with either 0.05 nmol or 1.0 nmol per mouse. The values are indicated as average ± SD obtained from each group of mice (n = 3−6) and listed as percentage of injected activity per gram tissue [% IA/g].

**[^{177}Lu]Lu-PSMA-617**						
	**4 h p.i.**		**24 h p.i.**		**48 h p.i.**	
	**0.05 nmol**	**1.0 nmol**	**0.05 nmol**	**1.0 nmol**	**0.05 nmol**	**1.0 nmol**
Blood	<0.10	<0.10	<0.10	<0.10	<0.10	<0.10
Heart	<0.10	<0.10	<0.10	<0.10	<0.10	<0.10
Lung	0.10 ± 0.03	<0.10	<0.10	<0.10	<0.10	<0.10
Spleen	0.30 ± 0.10	<0.10	<0.10	<0.10	<0.10	<0.10
Kidneys	14 ± 9	2.9 ± 0.6	0.86 ± 0.13	0.67 ± 0.18	0.46 ± 0.06	0.31 ± 0.10
Stomach	0.16 ± 0.09	0.16 ± 0.21	<0.10	<0.10	<0.10	<0.10
Intestines	0.19 ± 0.14	<0.10	<0.10	<0.10	<0.10	<0.10
Liver	<0.10	<0.10	<0.10	<0.10	<0.10	<0.10
Muscle	<0.10	<0.10	<0.10	<0.10	<0.10	<0.10
Bone	<0.10	0.20 ± 0.22	<0.10	<0.10	<0.10	<0.10
Salivary glands	<0.10	<0.10	<0.10	<0.10	<0.10	<0.10
LNCaP Tumor	8.3 ± 3.4	5.3 ± 0.9	12 ± 4	4.2 ± 1.1	7.9 ± 1.5	3.7 ± 0.8
Tumor-to-blood	193 ± 53	147 ± 31	1200 ± 449	440 ± 87	1177 ± 257	896 ± 144
Tumor-to-liver	90 ± 27	61 ± 12	149 ± 40	74 ± 14	184 ± 29	60 ± 9
Tumor-to-kidney	0.71 ± 0.28	1.8 ± 0.2	14 ± 6	6.2 ± 0.2	18 ± 6	12 ± 2

4. Area under the curve (AUC) values over 48 h and $AUC_{0\rightarrow48h}$ ratios

Purpose: The areas under the curves (AUC) were calculated for the first 48 h after injection of the radioligands in order to be able to judge the changes in activity uptake in the tumor and kidneys as a consequence of the tumor model and the injected amount of ligand, respectively.

Methods: The time-activity curves over the first 48 h after injection were determined for the PC-3 PIP tumor, the LNCaP tumor as well as for the kidneys of each tumor mouse model based on non-decay-corrected time-dependent biodistribution data. For these calculations, the radioligand uptake in the tumors and the kidneys at t = 0 h p.i. was set to 0% IA/g. The time-activity curves were used to calculate the areas under the curves ($AUCs_{0\rightarrow48h}$) using GraphPad Prism software (version 8).

Results: The results are shown in Table S5. The AUC values of the kidney uptake were similar in both mouse models, but significantly increased after injection of the lower molar ligand amount (0.05 nmol/mouse) than after the higher molar ligand amount (1.0 nmol/mouse). An increased accumulation of radioligand injected at low molar amount was also observed in the LNCaP tumors. In the case of PC-3 PIP tumor, only [^{177}Lu]Lu-Ibu-DAB-PSMA but not [^{177}Lu]Lu-PSMA-617 showed a significantly higher uptake when injected at low molar ligand amount. In all settings, the $AUC_{0\rightarrow48h}$ values obtained for the PC-3 PIP tumors were significantly higher than for the LNCaP tumors irrespective of the applied radioligand (Table S5).

Table S5 Area under the curve (AUC) values based on non-decay-corrected biodistribution data over the first 48 h after injection of either 0.05 or 1.0 nmol of [^{177}Lu]Lu-Ibu-DAB-PSMA or [^{177}Lu]Lu-PSMA-617 in either PC-3 PIP/flu or LNCaP tumor-bearing mice. The values are indicated as average ± SE and listed as percentage of injected activity per gram tissue times the hour [% IA/g*h]. $AUC_{0\rightarrow48h}$ values were calculated based on 4 h, 24 h and 48 h p.i. biodistribution data.

**PC-3 PIP tumor mouse model[a]: $AUC_{0\rightarrow48h}$ [% IA/g*h]**				
**Radioligand**	**[^{177}Lu]Lu-Ibu-DAB-PSMA**		**[^{177}Lu]Lu-PSMA-617**	
Ligand amount	0.05 nmol	1.0 nmol	0.05 nmol	1.0 nmol
PC-3 PIP tumor	3027 ± 206	2166 ± 153	1836 ± 132	1676 ± 120
Kidneys	945 ± 97	388 ± 23	187 ± 55	62 ± 11
**LNCaP tumor mouse model[b] : $AUC_{0\rightarrow48h}$ [% IA/g*h]**				
**Radioligand**	**[^{177}Lu]Lu-Ibu-DAB-PSMA**		**[^{177}Lu]Lu-PSMA-617**	
Ligand amount	0.05 nmol	1.0 nmol	0.05 nmol	1.0 nmol
LNCaP tumor	721 ± 115	402 ± 38	408 ± 69	179 ± 20
Kidneys	815 ± 103	349 ± 21	182 ± 94	50 ± 7

[a] PC-3 PIP tumors were grown in female mice; data of PC-3 flu tumors are not shown as the uptake was on background levels
[b] LNCaP tumors were grown in male mice.

5. SPECT/CT imaging studies

Purpose: The SPECT/CT imaging studies were performed with PC-3 PIP/flu and LNCaP tumor-bearing mice injected with two different quantities of [^{177}Lu]Lu-Ibu-DAB-PSMA or [^{177}Lu]Lu-PSMA-617 in order to visualize the impact of the tumor mouse model and the injected radioligand amount.

Methods: The methods of the SPECT/CT imaging studies are reported in the main article.

Results: The results are described in the main article and shown in Fig. 5, Fig. S1 and Fig. S2.

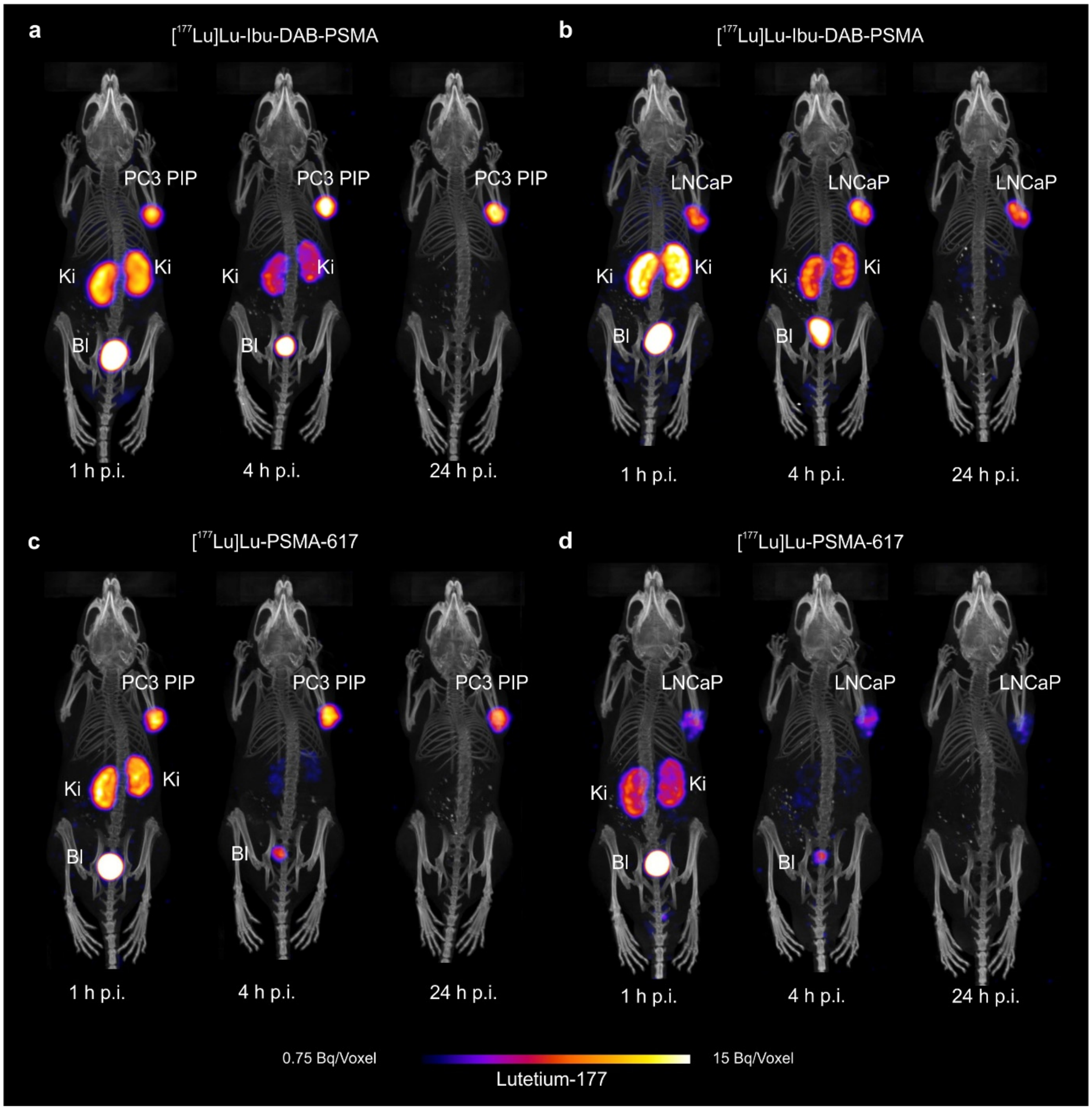

Fig. S1 SPECT/CT images obtained after injection of the radioligands (8 MBq; 0.05 nmol per mouse) shown as maximum intensity projections (MIPs). (**a**) Images of PC-3 PIP/flu-tumor-bearing mice after injection of [^{177}Lu]Lu-Ibu-DAB-PSMA; (**b**) Images of LNCaP tumor-bearing mice after injection of [^{177}Lu]Lu-Ibu-DAB-PSMA; (**c**) Images of PC-3 PIP/flu-tumor-bearing mice after injection of [^{177}Lu]Lu-PSMA-617; (**d**) Images of LNCaP tumor-bearing mice after injection of [^{177}Lu]Lu-PSMA-

617. PC-3 PIP = PSMA-transfected PC-3 human tumor xenograft; LNCaP = PSMA-positive human prostate cancer xenograft; Ki = kidney; Bl = urinary bladder

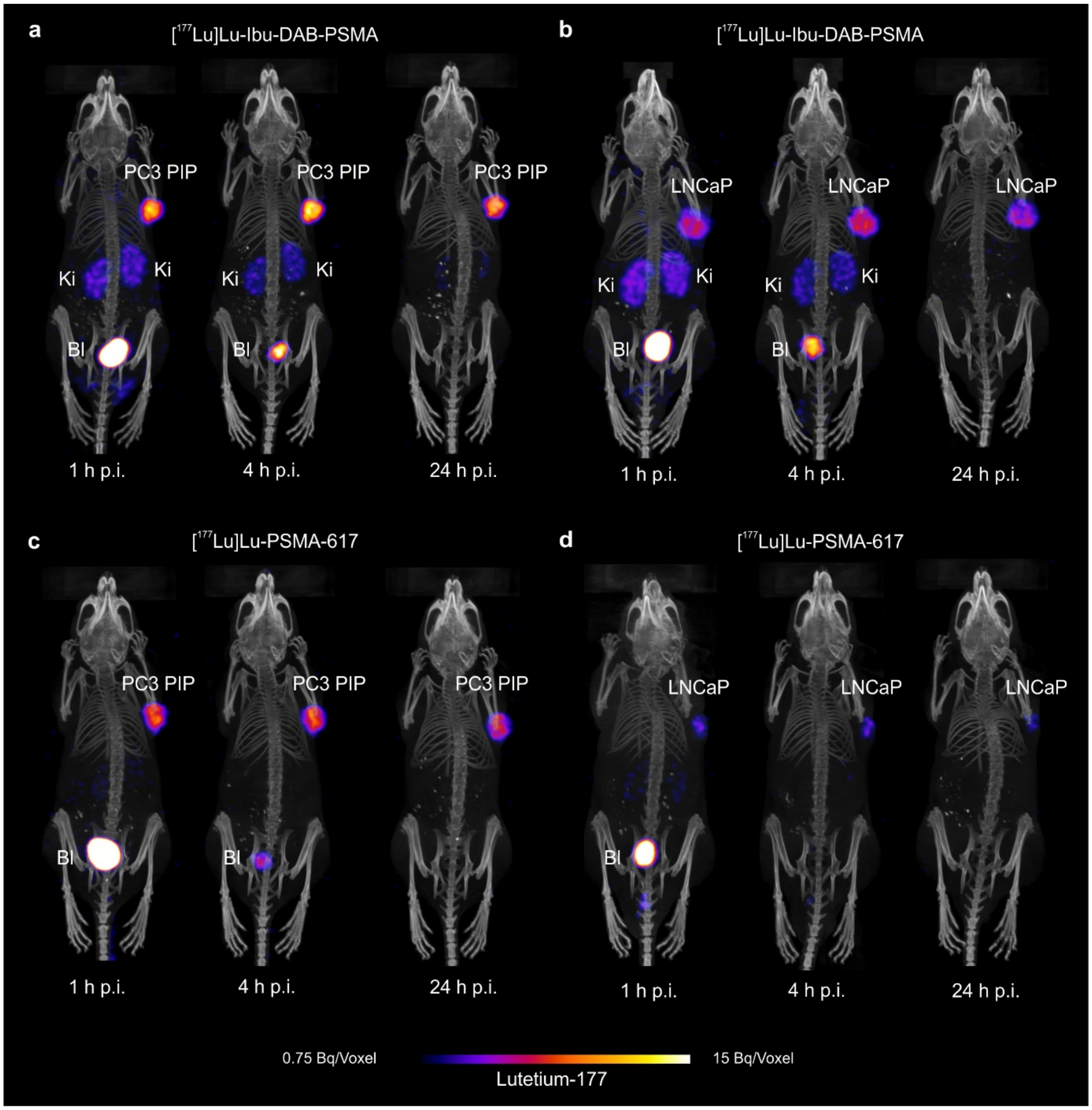

Fig. S2 SPECT/CT images obtained after injection of the radioligands (8 MBq; 1.0 nmol per mouse) shown as maximum intensity projections (MIPs). (**a**) Images of PC-3 PIP/flu-tumor-bearing mice after injection of [^{177}Lu]Lu-Ibu-DAB-PSMA; (**b**) Images of LNCaP tumor-bearing mice after injection of [^{177}Lu]Lu-Ibu-DAB-PSMA; (**c**) Images of PC-3 PIP/flu-tumor-bearing mice after injection of [^{177}Lu]Lu-PSMA-617; (**d**) Images of LNCaP tumor-bearing mice after injection of [^{177}Lu]Lu-PSMA-617. PC-3 PIP = PSMA-transfected PC-3 human tumor xenograft; LNCaP = PSMA-positive human prostate cancer xenograft; Ki = kidney; Bl = urinary bladder

References

1. Chang SS, Reuter VE, Heston WD, Bander NH, Grauer LS, Gaudin PB. Five different anti-prostate-specific membrane antigen (PSMA) antibodies confirm PSMA expression in tumor-associated neovasculature. Cancer Res. 1999;59:3192-8.
2. Liu C, Hasegawa K, Russell SJ, Sadelain M, Peng KW. Prostate-specific membrane antigen retargeted measles virotherapy for the treatment of prostate cancer. Prostate. 2009;69:1128-41. doi:10.1002/pros.20962.
3. Wu P, Kudrolli TA, Chowdhury WH, Liu MM, Rodriguez R, Lupold SE. Adenovirus targeting to prostate-specific membrane antigen through virus-displayed, semirandom peptide library screening. Cancer Res. 2010;70:9549-53. doi:10.1158/0008-5472.CAN-10-1760.
4. Choy CJ, Ling X, Geruntho JJ, Beyer SK, Latoche JD, Langton-Webster B, et al. ^{177}Lu-labeled phosphoramidate-based PSMA inhibitors: the effect of an albumin binder on biodistribution and therapeutic efficacy in prostate tumor-bearing mice. Theranostics. 2017;7:1928-39. doi:10.7150/thno.18719.
5. Wang Z, Tian R, Niu G, Ma Y, Lang L, Szajek LP, et al. Single low-dose injection of Evans blue modified PSMA-617 radioligand therapy eliminates prostate-specific membrane antigen positive tumors. Bioconjug Chem. 2018;29:3213-21. doi:10.1021/acs.bioconjchem.8b00556.
6. Umbricht CA, Benesova M, Schibli R, Müller C. Preclinical development of novel PSMA-targeting radioligands: modulation of albumin-binding properties to improve prostate cancer therapy. Mol Pharm. 2018;15:2297-306. doi:10.1021/acs.molpharmaceut.8b00152.
7. Deberle LM, Benesova M, Umbricht CA, Borgna F, Büchler M, Zhernosekov K, et al. Development of a new class of PSMA radioligands comprising ibuprofen as an albumin-binding entity. Theranostics. 2020;10:1678-93. doi:10.7150/thno.40482.
8. Horoszewicz JS, Leong SS, Kawinski E, Karr JP, Rosenthal H, Chu TM, et al. LNCaP model of human prostatic carcinoma. Cancer Res. 1983;43:1809-18.
9. Benesova M, Schäfer M, Bauder-Wüst U, Afshar-Oromieh A, Kratochwil C, Mier W, et al. Preclinical evaluation of a tailor-made DOTA-conjugated PSMA inhibitor with optimized linker moiety for imaging and endoradiotherapy of prostate cancer. J Nucl Med. 2015;56:914-20. doi:10.2967/jnumed.114.147413.
10. Weineisen M, Schottelius M, Simecek J, Baum RP, Yildiz A, Beykan S, et al. ^{68}Ga- and ^{177}Lu-labeled PSMA I&T: optimization of a PSMA-targeted theranostic concept and first proof-of-concept human studies. J Nucl Med. 2015;56:1169-76. doi:10.2967/jnumed.115.158550.
11. Kelly JM, Amor-Coarasa A, Nikolopoulou A, Wustemann T, Barelli P, Kim D, et al. Dual-target binding ligands with modulated pharmacokinetics for endoradiotherapy of prostate cancer. J Nucl Med. 2017;58:1442-9. doi:10.2967/jnumed.116.188722.
12. Kelly J, Amor-Coarasa A, Ponnala S, Nikolopoulou A, Williams C, Jr., Schlyer D, et al. Trifunctional PSMA-targeting constructs for prostate cancer with unprecedented localization to LNCaP tumors. Eur J Nucl Med Mol Imaging. 2018. doi:10.1007/s00259-018-4004-5.
13. Kuo HT, Merkens H, Zhang Z, Uribe CF, Lau J, Zhang C, et al. Enhancing treatment efficacy of ^{177}Lu-PSMA-617 with the conjugation of an albumin-binding motif: preclinical dosimetry and endoradiotherapy studies. Mol Pharm. 2018;15:5183-91. doi:10.1021/acs.molpharmaceut.8b00720.
14. Kuo HT, Lin KS, Zhang Z, Uribe CF, Merkens H, Zhang C, et al. Novel ^{177}Lu-labeled albumin-binder-conjugated PSMA-targeting agents with extremely high tumor uptake and enhanced tumor-to-kidney absorbed dose ratio. J Nucl Med. 2020. doi:10.2967/jnumed.120.250738.
15. Benesova M, Umbricht CA, Schibli R, Müller C. Albumin-binding PSMA ligands: optimization of the tissue distribution profile. Mol Pharm. 2018;15:934-46. doi:10.1021/acs.molpharmaceut.7b00877.
